# Initiating aerobic exercise with low glycogen content reduces markers of myogenesis but not mTORC1 signaling

**DOI:** 10.1186/s12970-021-00455-z

**Published:** 2021-07-10

**Authors:** Lee M. Margolis, Marques A. Wilson, Claire C. Whitney, Christopher T. Carrigan, Nancy E. Murphy, Adrienne Hatch-McChesney, Stefan M. Pasiakos

**Affiliations:** Military Nutrition Division, U.S. Army Research Institute of Environmental Medicine, 10 General Greene Avenue, Bldg. 42, Natick, MA 01760 USA

**Keywords:** High fat, High carbohydrate, Muscle regeneration, Endurance exercise

## Abstract

**Background:**

The effects of low muscle glycogen on molecular markers of protein synthesis and myogenesis before and during aerobic exercise with carbohydrate ingestion is unclear. The purpose of this study was to determine the effects of initiating aerobic exercise with low muscle glycogen on mTORC1 signaling and markers of myogenesis.

**Methods:**

Eleven men completed two cycle ergometry glycogen depletion trials separated by 7-d, followed by randomized isocaloric refeeding for 24-h to elicit low (LOW; 1.5 g/kg carbohydrate, 3.0 g/kg fat) or adequate (AD; 6.0 g/kg carbohydrate, 1.0 g/kg fat) glycogen. Participants then performed 80-min of cycle ergometry (64 ± 3% VO_2peak_) while ingesting 146 g carbohydrate. mTORC1 signaling (Western blotting) and gene transcription (RT-qPCR) were determined from vastus lateralis biopsies before glycogen depletion (baseline, BASE), and before (PRE) and after (POST) exercise.

**Results:**

Regardless of treatment, p-mTORC1^Ser2448^, p-p70S6K^Ser424/421^, and p-rpS6^Ser235/236^ were higher (*P* < 0.05) POST compared to PRE and BASE. *PAX7* and *MYOGENIN* were lower (*P* < 0.05) in LOW compared to AD, regardless of time, while *MYOD* was lower (*P* < 0.05) in LOW compared to AD at PRE, but not different at POST.

**Conclusion:**

Initiating aerobic exercise with low muscle glycogen does not affect mTORC1 signaling, yet reductions in gene expression of myogenic regulatory factors suggest that muscle recovery from exercise may be reduced.

## Introduction

Reducing muscle glycogen through sustained aerobic exercise followed by low carbohydrate, high fat feeding increases fat oxidation and decreases carbohydrate oxidation during subsequent exercise bouts [1–4]. When aerobic exercise is initiated with low glycogen, adjustments in intramuscular molecular signaling modulate changes in substrate oxidation, compared to exercising when glycogen stores are high [5]. Low glycogen availability upregulates transcription of fatty acid translocase (FAT), carnitine palmitoyl transferase 1a (CPT1a) and hydroxyacyl-CoA dehydrogenase (HADHA), which govern fatty acid uptake, transport, and oxidation [6–9]. Concurrent with transcriptional changes in fatty acid metabolism are reductions in pyruvate dehydrogenase (PDH) activity which spares glycogen by reducing carbohydrate oxidation during exercise [10, 11]. These acute molecular adaptations to initiating aerobic exercise with low muscle glycogen persist even when carbohydrate is ingested during the exercise bout [12].

Though performing aerobic exercise with low glycogen results in molecular adaptations that facilitate increased fat oxidation [5], this practice may compromise muscle repair and recovery [13, 14]. Reliance on protein for oxidative purposes increases when aerobic exercise is initiated with low glycogen [13–15]. Increased use of protein for fuel appears to contribute to lower rates of muscle protein synthesis and blunted activation of the mechanistic target of rapamycin complex 1 (mTORC1) signaling cascade following exercise [13, 16, 17]. Maintaining low glycogen by adhering to a low carbohydrate, high-fat diet may also contribute to impaired muscle repair and recovery after aerobic exercise [18, 19]. Increased concentrations of free fatty acids using lipid infusions in humans [18] or lipid-enriched media in cell culture models [19] decreases muscle protein synthesis and expression of myogenic regulatory factors, *PAX7*, *MYOD*, and *MYOGENIN*, thereby impairing myotube formation. Suppressed myogenesis in cell culture models suggests that increased fatty acid availability reduces muscle repair and regeneration. To the best of our knowledge, this posited mechanism has not been confirmed in humans. Furthermore, whether consuming carbohydrate during exercise initiated with low glycogen mediates the potential negative effects on myogenesis by stimulating AKT to upregulate mTORC1 signaling is unclear [20, 21].

The objective of this study was to examine the effects of glycogen availability on mTORC1 signaling and marks of myogenesis before and after a bout of aerobic exercise. We manipulated glycogen availability for those experiments using a glycogen depleting bout of cycle ergometry followed by high carbohydrate, low-fat or low carbohydrate, high-fat diets for the 24 h before the experiments. Additionally, this study examined the impact of consuming carbohydrate during a subsequent bout steady-state aerobic exercise initiated with low glycogen on post exercise mTORC1 signaling and markers of myogenesis. We hypothesized that mTORC1 signaling and myogenesis would be lower when aerobic exercise was initiated with low compared to adequate glycogen.

## Materials and methods

### Participants

Twelve healthy, non-obese, recreationally active men between the ages of 18–39 years were enrolled to participate in this randomized, crossover study after providing informed, written consent. Individuals were excluded from the study if they were not in good health (metabolic or cardiovascular abnormalities, gastrointestinal disorders such as kidney disease, diabetes, and cardiovascular disease or were taking medications, such as stains, corticosteroids or diabetes medication that may affect macronutrient metabolism), refused to abstain from alcohol, nicotine, and dietary supplements during the study, had musculoskeletal injuries that compromised their ability to exercise, or donated blood within 8 weeks of beginning the study. Treatment order randomization was done using a random number generator. Participants in this study were part of a larger investigation that also aimed to assess the impact of glycogen content on microRNA expression and rates of exogenous glucose oxidation during steady-state aerobic exercise (Trial registration: *Carbohydrate Availability and microRNA Expression*, *registered 15 August 2017*, https://clinicaltrials.gov/ct2/show/NCT03250234). Results from this separate objective are reported elsewhere [12]. Data are reported on 11 of the 12 enrolled participants because we were unable to collect a baseline muscle biopsy on one participant. This study was approved by the Institutional Review Board at the US Army Medical Research and Development Command (MRDC, Fort Detrick, MD) and data collection took place at the US Army Research Institute of Environmental Medicine (USARIEM, Natick, MA), between August 2017 to May 2018.

Pre-study participant characteristics, including height (Seritex, Inc., Carlstadt, NJ, USA), body mass (WB-110A, Tanita, Tokyo, Japan), and body composition (dual energy x-ray absorptiometry, DPX-IQ, GE Lunar Corporation, Madison, WI, USA), are reported in Table 1. Peak oxygen uptake (V̇O_2peak_) was determined using a progressive-intensity cycle ergometer (Lode, BV, Netherlands) test and an indirect, open circuit respiratory system (True Max 2400, Parvomedics, Sandy, Utah, USA). Exercise intensities for protocol days were based on volunteers V̇O_2peak_.

**Table 1:** Participant characteristics

Characteristics	
Age (yrs)	21 ± 4
Height (m)	1.8 ± 0.1
Weight (kg)	83 ± 11
Body Mass Index (kg/m^2^)	26 ± 2
Fat mass (kg)	19 ± 10
Fat-free mass (kg)	63 ± 9
V̇O_2peak_ (mL/min/kg)	44 ± 4

Values are mean ± SD, n = 11

### Experimental design

**Fig. 1:**
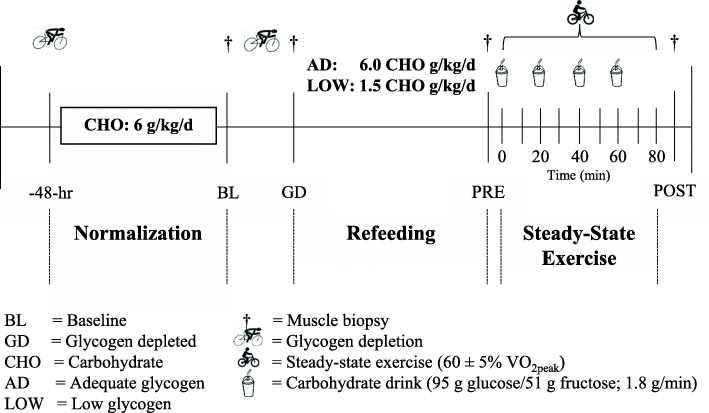
Experimental design

To normalize muscle glycogen between study arms, participants completed a glycogen depletion (Lode, BV, Netherlands) protocol 48-h prior to testing (Fig. 1), as previously described [12]. Participants completed 2-min of high-intensity cycling (work period) at 90% V̇O_2_peak, followed by a 2-min recovery period cycling at 50% V̇O_2peak_ [2]. This work-to-recovery ratio was maintained until the participant was no longer able to complete 2-min of cycling at 90% V̇O_2peak_. Cycling intensity during the work period was progressively lowered to 80%, 70%, and 60% V̇O_2peak_ when the participant was unable to complete 2-min of cycling at the given workload. Once the participant could not complete 2-min of cycling at 60% V̇O_2peak_, exercise was stopped. The recovery period was maintained at 50% V̇O_2peak_. Participants performed two practice sessions to ensure they were familiar with the protocol before testing.

Following glycogen depletion, dietary intake was controlled to normalize glycogen status before experimental days. All food and beverages (except water, which was allowed ad libitum) were provided during this glycogen normalization phase by study dietitians, who designed and prepared meals, derived from Meals, Ready-to-Eat (MRE; Ameriqual, Evansville, IN, USA) combat rations and commercially available food items. Participants returned all food and beverage wrappers and containers to dietitians to confirm intake. During the glycogen normalization phases, intakes were the same for LOW and AD; averaging 5.7 ± 0.6 g/kg/d carbohydrate, 1.2 ± 0.1 g/kg/d protein, and 1.0 ± 0.1 g/kg/d fat.

Following a 10 h overnight fast, a baseline (BL) muscle biopsy was taken from the vastus lateralis after glycogen normalization. Participants then completed the same glycogen depletion protocol as performed during the normalization phase. Glycogen depletion exercise time (LOW: 84 ± 25, AD: 88 ± 24 min) and intensity (mean power; LOW: 164 ± 26, AD: 161 ± 25 watts) were similar between treatments [12]. After glycogen depletion (GD), a second muscle biopsy was taken to confirm reductions in glycogen stores using an endpoint colorimetric assay (Cat# MAK016; Sigma-Aldrich, St. Louis, MO, USA) as reported elsewhere [12]. There was no difference in glycogen content at BL (LOW; 467 ± 95, AD; 472 ± 109 µmol/kg muscle dry weight) or after GD (LOW; 207 ± 99, AD; 210 ± 145 µmol/kg muscle dry weight) between LOW and AD [12]. Participants were then fed an isocaloric diet for the remainder of the day to elicit LOW (3081 ± 374 kcal/d, 1.5 ± 0.1 g/kg/d carbohydrate, 1.3 ± 0.5 g/kg/d protein, and 3.0 ± 0.5 g/kg/d fat) or AD (3086 ± 347 kcal/d, 6.0 ± 0.2 g/kg/d carbohydrate, 1.2 ± 0.5 g/kg/d protein, and 1.0 ± 0.5 g/kg/d fat) glycogen stores.

Participants returned to the laboratory the following day after a 10 h overnight fast. Resting metabolic rate (RMR) was measured to assess rested/fasted substrate oxidation before exercise (PRE) using an open-circuit indirect calorimetry (Parvo Medics). Participants rested in the supine position for ~ 30 min before expired air was collected using an acrylic hood. The test was discontinued when 20 min of steady-state V̇O_2_ and V̇CO_2_ were recorded. After the RMR testing protocol, a pre-exercise (PRE) muscle biopsy was taken. Participants then consumed 550 mL of the carbohydrate drink immediately before starting the exercise bout. Participants then began cycling for 80 min at their target V̇O_2_ (LOW; 65 ± 4, AD; 64 ± 3% V̇O_2peak_)_._ Participants consumed 300 mL of the carbohydrate drink at 20, 40, and 60-min during exercise. Total carbohydrate ingested was 146 g (95 g glucose + 51 g fructose), consumed at an average ingestion rate of 1.8 g/min. The carbohydrate drink was prepared by the Combat Feeding Directorate (Natick, MA, USA) and contained corn-derived crystalline fructose (KRYSTAR® 30 0, Tate and Lyle Sugars, London, UK), maltodextrin (MALTRIN QD® M500, Grain Processing Corporation, Muscatine, IA, USA) and dextrose (CERELOSE®, Ingredion, Westchester, IL, USA). Nutrient content was confirmed before use (Eurofins Food Chemistry Testing Madison, Inc, Madison, WI, USA). During exercise, respiratory gas exchange (Parvo Medics) was measured at 0, 15, 30, 45, 60, and 75-min to assess substrate oxidation. A final biopsy was taken at the end of exercise (POST). Per study design [12], PRE and POST glycogen was lower in LOW than AD (Table 2). Following a minimum 7 day washout period, participants returned to the laboratory to complete the second arm of the study.

**Table 2:** Muscle glycogen and substrate oxidation. Adapted from Margolis et al. [12]

	Group	Steady-state cycle ergometry		
		PRE	DURING	POST
Glycogen (µmol/kg dry muscle wt)	LOW	217 ± 103^**+**^	–	137 ± 131^**+**^
	AD	396 ± 70	–	229 ± 94^‡^
Carbohydrate oxidation (g/min)	LOW	0.04 ± 0.03^**+**^	1.59 ± 0.40^**+**^	–
	AD	0.12 ± 0.02	2.03 ± 0.36	–
Fat oxidation (g/min)	LOW	0.12 ± 0.02^**+**^	0.55 ± 0.10^**+**^	–
	AD	0.09 ± 0.02	0.38 ± 0.13	–

Values mean ± SD, n = 11

^+^LOW different than AD; *P* < 0.05. ^‡^POST different than PRE; *P* < 0.05

### Substrate oxidation

Resting carbohydrate and fat oxidation was calculated as [22]:

$$ {\text{Fat}}\;{\text{oxidation }}\left( {{\text{g}}/{\text{min}}} \right) = 1.67 \times {\dot{\text{V}}\text{O}}_{2} \left( {{\text{L}}/{\text{min}}} \right){-} 1.67 \times {\dot{\text{V}}\text{CO}}_{2} \left( {{\text{L}}/{\text{min}}} \right) $$

$$ {\text{Total}}\;{\text{carbohydrate}}\;{\text{oxidation}}\left( {{\text{g}}/{\text{min}}} \right) = 4.55 \times {{\dot{\text{V}}}\text{CO}}_{{\text{2}}} \left( {{\text{L}}/{\text{min}}} \right){-}3.21 \times {{\dot{\text{V}}}\text{O}}_{2} \left( {{\text{L}}/{\text{min}}} \right) $$

Exercise carbohydrate and fat oxidation were calculated [23]:

$$ {\text{Fat}}\;{\text{oxidation}}\left( {{\text{g}}/{\text{min}}} \right) = 1.695 \times {{\dot{\text{V}}}\text{O}}_{2} \left( {{\text{L}}/{\text{min}}} \right){-}1.701 \times {\dot{\text{V}}\text{CO}}_{2} \left( {{\text{L}}/{\text{min}}} \right) $$

$$ {\text{Total}}\;{\text{carbohydrate}}\;{\text{oxidation}}\left( {{\text{g}}/{\text{min}}} \right) = 4.585 \times {\dot{\text{V}}\text{CO}}_{2} \left( {{\text{L}}/{\text{min}}} \right){-}3.226 \times {\dot{\text{V}}\text{O}}_{2} \left( {{\text{L}}/{\text{min}}} \right). $$

Substrate oxidation data during exercise were previously reported [12], but are briefly presented in Table 2 of this report to highlight differences in fuel use during exercise between the two treatments. Comparison of resting substrate oxidation data between LOW and AD has not been previously published.

### Muscle biopsies

Percutaneous muscle biopsies were conducted on the vastus lateralis using a 5-mm Bergstrom needle with manual suction while the participant was under local anesthesia (1% lidocaine). Muscle biopies were conducted immediately before and after the glycogen depletion protocol from a single incision made in a randomly selected leg. Muscle biopsies were also conducted before and after steady-state cylcing in the contralateral leg from a single incision. The average muscle sample weight was 110 mg. Immediately after being weighed, muscle samples were snap frozen in liquid nitrogen. At the conclusion of study muscle samples were cut under liquid nitrogen and aliquoted for assessment of muscle glycogen content, activity assays, intracellcular signaling, and mRNA expression for the current and our previously published manuscript [12].

### Intracellular signaling

Phosphorylation status and total protein content were determined using Western blotting. Muscle samples (~ 20 mg) were homogenized in ice-cold homogenization buffer (1:10 w/v) that contained 50 mM Tris–HCl (pH 7.5), 5 mM Na-pyrophosphate, 50 mM NaF, 1 mM.

EDTA, 1 mM EGTA, 10% glycerol (v/v), 1% Triton X-100, 1 mM DTT, 1 mM benz-amidine, 1 mM PMSF, 10 mg/mL trypsin inhibitor, and 2 mg/mL aprotinin. Samples were homogenized using a TissueLyser II with a 5-mm steel bead (Qiagen, Valencia, CA, USA). Homogenates were centrifuged for 15 min at 10,000×*g* at 4 °C. Supernatant (lysate) was collected and protein concentrations were determined using 660 nm Protein Assay (ThermoFisher, Waltham, MA, USA). Muscle lysates were solubilized in Laemmli buffer, with equal amounts of total protein loaded (15 µg) and separated by SDS-PAGE using precast Tris–HCl gels (Bio-Rad, Hercules, CA, USA). Proteins were transferred to polyvinylidene fluoride membranes and exposed to commercially available primary antibodies specific to p-AMPK^Thr72^, p-AKT^Thr473^, p-mTOR^Ser2448^, p-p70S6K^Thr424/421^, p-rpS6^Ser235/236^, AMPK, AKT, mTOR, p70S6K, and rpS6 (Cell Signaling Technology, Danvers, MA) overnight at 4 °C. Secondary antibody (anti‐rabbit IgG conjugate with horseradish peroxidase; Cell Signaling Technology) and chemiluminescent reagent (Pierce Biotechnology, Rockford, IL) were applied to label primary antibodies. Blots were quantified using a phosphoimager (ChemiDoc XRS; Bio‐Rad) and Image Lab software (Bio‐Rad). Heat shock protein 90 (HSP90) was used to confirm equal amounts of protein were loaded per well. Phosphorylation status was normalized to its total protein. Data presented as fold change relative to BL phosphorylation for each group.

### mRNA expression

Total RNA was isolated in ~ 20 mg muscle samples using TRIzol reagent (Thermo Fisher). RNA quantity and quality were assessed using a Nanodrop ND-2000 spectrophotometer (Nanodrop, Wilmington, DE, USA). For mRNA analysis, equal amounts of total RNA (500 ng) were reverse-transcribed using the high-capacity cDNA reverse transcription (RT) kit (Applied Biosystems, Foster City, CA, USA). Reverse transcription was conducted in a T100™ Thermal Cycler (Bio-Rad). Amplifications were performed using a StepOnePlus Real-Time PCR System (Applied Biosystems). Samples were run in 10 µL reactions in duplicate using TaqMan® fast advanced master mix and commercially available TaqMan® probes (*PAX7, MYOD, MYOGENIN;* Applied Biosystems). All mRNA were normalized to *B2M*. Fold change for mRNA PRE and POST steady-state exercise were calculated using the ΔΔ cycle threshold (ΔΔCT) method [24] and expressed relative to individual BL values.

### Statistical analysis

Normality for all data was confirmed using Shapiro–Wilk tests for dependent variables. Paired t-tests were used to assess differences in substrate oxidation at rest and during exercise between treatments (LOW vs. AD). Mixed-model repeated measures ANOVA was used to assess glycogen, phosphorylation status, and mRNA expression for effects of time, treatment, and their interactions. Bonferroni adjustments for multiple comparisons were performed if significant interactions were observed. All data are presented as mean ± SD. The α level for significances was set at *P* < 0.05. Data were analyzed using IBM SPSS Statistics for Windows Version 26.0 (IBM Corp. Armonk, NY, USA).

## Results

### Intracellular signaling

**Fig. 2:**
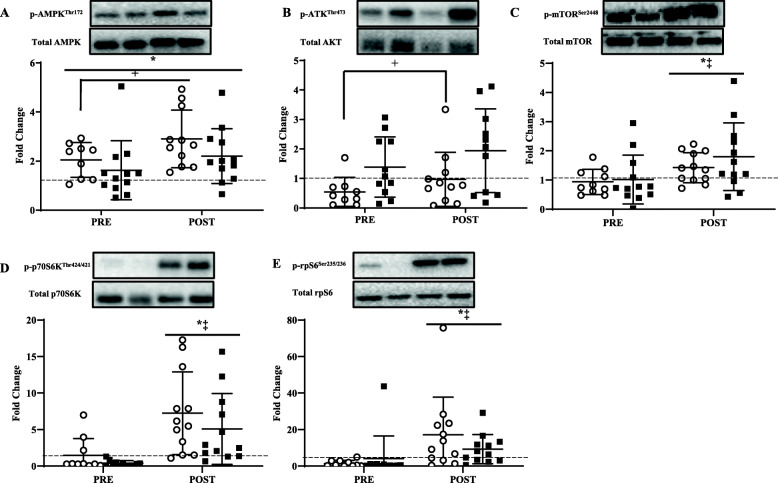
Fold change before (PRE) and after (POST) steady-state for p-AMPK^Thr172^ (**A**), p-AKT^Thr473^ (**B**), p-mTOR^Ser2448^ (**C**), p-p70S6K^Thr424/421^ (**D**), and p-rpS6^Ser235/236^ (**E**) relative to baseline (BL) values (dotted line) for low (LOW; circle) or adequate (AD; black square) glycogen status. Values are mean ± SD, n = 11. *Different than BL, *P* < 0.05. ^‡^Different than PRE, *P* < 0.05. ^+^Different than AD, *P* < 0.05

Independent of time, p-AMPK^Thr172^ was higher (*P* < 0.05) and p-AKT^Thr473^ was lower (*P* < 0.05) in LOW than AD (Fig. 2A, B).Regardless of treatment, p-AMPK^Thr172^ was higher (*P* < 0.05) at PRE and POST compared to BL. Independent of treatment, POST p-mTOR^Ser2448^, p-p70S6K^Thr424/421^, and p-rpS6^Ser235/236^ were higher (*P* < 0.05) than BL and PRE (Fig. 2C–E).

### mRNA expression

**Fig. 3:**
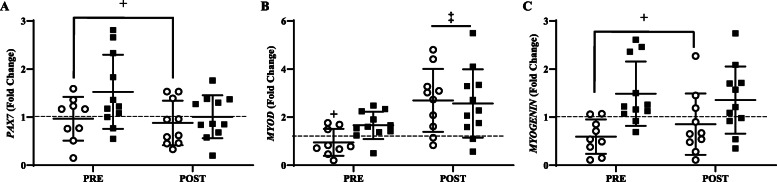
Fold change before (PRE) and after (POST) steady-state for *PAX7* (**A**), *MYOD* (**B**), and *MYOGENIN* (**C**) relative to baseline (BL) values (dotted line) for low (LOW; circle) or adequate (AD; black square) glycogen status. Values are mean ± SD, n = 11. *Different than BL, *P* < 0.05. ^‡^Different than PRE, *P* < 0.05. ^+^Different than AD, *P* < 0.05

Independent of time, *PAX7* was lower (*P* < 0.05) in LOW than AD (Fig. 3A). PRE *MYOD* was lower (*P* < 0.05) in LOW than AD (Fig. 3B). POST *MYOD* in LOW and AD were higher (*P* < 0.05) than PRE, with no difference between group. Independent of time, *MYOGENIN* was lower (*P* < 0.05) in LOW than AD (Fig. 3C).

## Discussion

The primary results from this study were that despite differences in AMPK and AKT phosphorylation, initiating aerobic exercise with low glycogen did not affect downstream mTORC1 signaling compared to adequate glycogen. Though no differences in mTORC1 signaling were observed, lower glycogen at the onset of exercise reduced the expression of myogenic regulatory factors, *PAX7*, *MYOD*, and *MYOGENIN*. Reductions in myogenesis may indicate lower post-exercise muscle recovery.

The most novel finding from the current study was that performing aerobic exercise with low muscle glycogen content resulted in lower *PAX7* and *MYOGENIN* expression before and after exercise compared to adequate glycogen. Additionally, low glycogen status reduced *MYOD* expression under resting fasted conditions compared to adequate glycogen. However, following aerobic exercise and carbohydrate consumption, MYOD expression was increased compared to pre aerobic exercise, and was not different between groups. These mediators of myogenesis are integral to the activation of myogenic precursor cells (e.g., satellite cells), facilitating proliferation and differentiation of myoblasts into myotubes; a primary function required for muscle repair and regeneration [25, 26]. As such, reductions in the transcription of these myogenic-related genes may impede muscle recovery from exercise [27]. It appears that differences in *PAX7*, *MYOD*, and *MYOGENIN* between LOW and AD were primarily driven by lower expression at PRE. The PRE measurement was taken 24 h after participants completed glycogen depleting exercise followed by high carbohydrate, low-fat (63% carbohydrate or 24% fat) or low carbohydrate, high-fat (16% carbohydrate and 71% fat) refeeding diet to achieve AD or LOW muscle glycogen, respectively. Whether low glycogen availability, increased fat intake, or a combination of both caused lower myogenesis in LOW compared to AD is not clear, because exercise and diet manipulation were used to manipulate glycogen availability in both treatments.

Given the differences in AKT between treatments, we hypothesize that lower phosphorylation of AKT in LOW facilitated lower myogenic regulatory factor expression compared to AD. AKT is an important upstream regulator of myogenesis [28, 29]. Overexpression of AKT in C2C12 myoblasts induces myogenic differentiation by increasing myogenic regulator factor expression to simulate myotube formation, while inhibition of AKT decreases myogenesis [28–30]. In the current study, alterations in myogenesis via AKT were likely through downstream activation of GSK-3β [31]. GSK-3β regulates glycogen stores through inhibition of glycogen synthase activity, thereby blunting the conversion of glucose to glycogen within muscle [32]. Lower phosphorylation of AKT, similar to that observed in the present study, increases GSK-3β activity resulting in reduced glycogen synthesis within skeletal muscle [21]. Beyond regulation of glycogen content, in vitro experiments have reported that GSK-3β is an AKT intermediate and regulator of myogenesis [31]. Increased insulin-mediated phosphorylation of AKT deactivates GSK-3β, resulting in increased expression of myogenic regulatory factors to facilitate myogenesis [31, 33]. We suspect higher phosphorylation of AKT in AD PRE and POST mediated the higher expressions of *PAX7*, *MYOD*, and *MYOGENIN* compared to LOW.

Increased fat intake post exercise may also have contributed to lower expression of myogenic regulatory factors in LOW compared to AD. Differentiating C2C12 myoblasts in medium that contained fatty acids resulted in reduced myotube formation compared to control [19]. Reduced myotube formation was the result of lower *PAX7*, *MYOD*, and *MYOGENIN* in the lipid-enriched media compared to control [19]. In addition to lower expression of myogenic regulatory factors, the same in vitro experiment [19] reported higher expression of CPT1 and FAT; genes associated with adipogenesis. Those observations agree with our previous findings, where we reported increased expression of molecular regulators of fatty acid uptake and transport with higher fat intake [12]. Together these results may suggest that acute increases in fatty acid availability upregulates expression of genes associated with adipogenesis while genes regulating myogenesis are reduced.

There was no effect of glycogen content on phosphorylation status of the mTOR, p70S6K, and rpS6. Several previous human studies have examined the effect of glycogen content on muscle protein synthesis and anabolic signaling [13, 16, 17, 34]. Some report either a decrease [13, 17] or no difference [16, 34] in muscle protein synthesis or mTORC1 signaling when initiating exercise with low glycogen content. The discrepancies between studies may be partially explained by differences in exercise modality (resistance vs. aerobic vs. high intensity interval exercise), energy state (energy balance vs. energy deficit), and fed state (fed vs. fasted). Another factor that may contribute to variable results across studies is the severity by which muscle glycogen is lowered. Hammond et al. [16] hypothesized that muscle glycogen must be < 100 µmol/kg dry weight to negatively affect the anabolic response to exercise. Indeed, Impey et al. [17] reported lower p70S6K activity post exercise when muscle glycogen content was reduced to < 100 µmol/kg dry weight, and reported that the severity of the decline in p70S6K activity was associated with glycogen content. Our data agree with this hypothesis, as mTORC1 signaling was maintained when aerobic exercise was initiated with muscle glycogen concentrations of ~ 200 µmol/kg dry weight in LOW compared to ~ 400 µmol/kg dry weight in AD.

Results from this study provide novel insight into the effects of glycogen availability on post-exercise mTORC1 signaling and myogenesis in muscle. However, a limitation of our study was the lack of serial blood sampling during exercise. Not assessing the changes in glucose, insulin, and free-fatty acids concentrations during exercise prohibits us from examining the potential impact of possible differences in circulating substrates between treatments on skeletal muscle adaptations. Additionally, due to limited sample availability we were not able to measure GSK-3β to assess if its phosphorylation status was in agreement with our AKT data. Though GSK-3β was not measured in the current study, it stands to reason that differences in glycogen content between treatments would be the result of differences in molecular pathways governing glycogen synthesis. Finally, results of the current study should be interpreted in the context in which they were collected. Our results show acute differences in markers of myogenesis when exercise is initiated with low or adequate glycogen stores. The impact of these acute signaling and transcriptional modifications on long term training adaptations effecting muscle mass, phenotype, and physical performance are unclear.

## Conclusion

In conclusion, initiating aerobic exercise with low muscle glycogen increased phosphorylation of AMPK and decreased phosphorylation of AKT compared to adequate glycogen. Despite differences in upstream targets, low glycogen content did not affect insulin-dependent mTORC1 signaling, but did result in reduced expression of myogenic regulatory factors. Lower expression in key myogenic regulator factors suggest that muscle recovery from exercise may be reduced when exercise is performed with low muscle glycogen.

## Data Availability

All extracted data are presented in this manuscript. The corresponding author may be contacted for any data requests or questions.

## Abbreviations

FAT: Fatty acid translocase
CPT1a: Carnitine palmitoyl transferase 1a
HADHA: Hydroxyacyl-CoA dehydrogenase
PDH: Pyruvate dehydrogenase
mTORC1: Mechanistic target of rapamycin complex 1
MRDC: Medical Research and Development Command
LOW: Low glycogen
AD: Adequate glycogen
BL: Baseline
PRE: Pre exercise
POST: Post exercise
